# Association of Pulse Pressure with Serum TNF-**α** and Neutrophil Count in the Elderly

**DOI:** 10.1155/2014/972431

**Published:** 2014-05-27

**Authors:** Eriko Yamada, Mika Takeuchi, Miki Kurata, Tsutomu Kazumi, Keisuke Fukuo

**Affiliations:** ^1^Postgraduate School of Food Sciences and Nutrition, Mukogawa Women's University, 6-46 Ikebiraki-cho, Nishinomiya, Hyogo 663-8558, Japan; ^2^Department of Food Sciences and Nutrition, School of Human Environmental Sciences, Mukogawa Women's University, 6-46 Ikebiraki-cho, Nishinomiya, Hyogo 663-8558, Japan; ^3^Research Institute for Nutrition Sciences, Mukogawa Women's University, 6-46 Ikebiraki-cho, Nishinomiya, Hyogo 663-8558, Japan; ^4^Diabetes Center, Myodani Hospital, 2350-2 Aza Nashihara, Myodani-cho, Tarumi-ku, Kobe, Hyogo 655-0852, Japan

## Abstract

*Aims.* Elevated pulse pressure (PP) has been reported to be a risk factor for type 2 diabetes in elderly patients with hypertension. *Methods.* Cross-sectional relationships of PP with known risk factors for type 2 diabetes and inflammatory markers were examined in 150 elderly community-dwelling women, 79 women (52.7%) of whom had hypertension. *Results.* Systolic blood pressure (standardized **β**, 0.775), log tumor necrosis factor-**α** (TNF-**α**, standardized **β**, 0.110), age (standardized **β**, 0.140), and neutrophil count (standardized **β**, 0.114) emerged as determinants of PP independent of high-sensitivity C-reactive protein, interleukin-6, monocyte count, plasminogen activator inhibitor-1, homeostasis model assessment of insulin resistance, HDL-cholesterol, and adiponectin (*R* ^2^ = 0.772). *Conclusions.* The present studies have demonstrated an independent association of higher PP with higher TNF-**α**, a marker of insulin resistance, and neutrophil count in community-living elderly women and suggest that insulin resistance and chronic low-grade inflammation may in part be responsible for the association between high PP and incident type 2 diabetes found in elderly patients with hypertension.

## 1. Background

Several studies have shown that elevations in pulse pressure (PP) are predictive of an increased risk of cardiovascular disease (CVD) [1, 2]. Although pulse pressure derives from the interaction of cardiac ejection (stroke volume) and the properties of arterial circulation (arterial stiffness and wave reflection), elevated PP is thought to be largely associated with increased arterial stiffness due to aging, arteriosclerosis, or both [3, 4]. Several recent studies have reported an association between insulin resistance, a hallmark of type 2 diabetes, and increased arterial stiffness [5, 6].

There is increasing evidence that low-grade inflammation is closely involved in the pathogenesis of type 2 diabetes [7]. Recently, brachial PP has been reported to be an independent risk factor for new-onset diabetes in high-risk Japanese hypertensive patients [8]. As PP is a predictor of CVD mainly over the age of 50 years [9] and PP is higher in women than in men [2], relationships between PP and a broad range of risk factors for type 2 diabetes including several biomarkers of insulin resistance, serum hepatic enzyme levels [10], adipokines [11], and inflammation markers have been studied in elderly women in the present study. As cystatin C has recently been reported as a risk factor for type 2 diabetes [12], we also examined the relationship between PP and the new marker for kidney function because reduced renal function and low grade-inflammation are associated with each other and are common in the elderly population [13].

## 2. Participants and Methods

We here show results of 150 out of 361 free-living elderly women whose details have been reported elsewhere [14] because the 150 women participated in the fasted condition and were not on antidiabetic drugs. Insulin resistance, a strong and well-known risk factor for type 2 diabetes, was assessed using homeostasis model, which requires fasting glucose and insulin as described below. Participants were residents in Nishinomiya City and were recruited as volunteers by local welfare commissioners from the city of Nishinomiya, Hyogo, Japan. Although 43, 9, and 58 women of 159 women (27.0%, 5.7%, and 36.5%, resp.) reported to be receiving statins and antidiabetic and antihypertensive drugs, respectively [14], detailed drug information was not available. Subjects with clinically diagnosed acute or chronic inflammatory diseases, endocrine, cardiovascular, hepatic, renal diseases, hormonal contraception, and unusual dietary habits were also excluded from the study. Because the aim of the present studies is to determine the relationships between PP and risk factors for type 2 diabetes, 9 participants on antidiabetic drugs were also excluded from the analysis. This research followed the tenets of the Declaration of Helsinki. The design of this study was approved by the Ethical Committees of Mukogawa Women's University and written informed consents were obtained from all participants.

Body weight and height were measured following standard procedures after an overnight fasting and BMI was calculated. Fat mass was measured using an impedance method (InBody 430, Biospace, Tokyo, Japan). Systolic and diastolic blood pressure (SBP and DBP, resp.) were measured using a fully automated sphygmomanometer (BP-203RV II, Colin, Tokyo, Japan) after participants had rested at least 5 min. The measurements were repeated after 2-3 min and the average of the measurements was used in analysis. PP was calculated as the difference between SBP and DBP in each participant. PP ≧ 65 mmHg was defined to be high as previously reported [1].

We evaluated routine chemical parameters, including liver enzymes, glucose, insulin, lipids, and lipoproteins, as previously reported [14, 15] and insulin resistance was evaluated using homeostasis model assessment (HOMA-IR) [16].

Adipokines and inflammatory markers were measured as previously reported [14, 15]. They included adiponectin, leptin, plasminogen activator inhibitor-1 (PAI-1), preheparin lipoprotein lipase (LPL), high-sensitivity C-reactive protein (hsCRP), interleukin-6 (IL-6), and tumor necrosis factor-1 (TNF-*α*). Peripheral leukocyte analyses included total leukocyte counts and differential percentages of neutrophils, monocytes, lymphocytes, eosinophils, and basophils using an automated cell counter (XE-2100 Hematology Alpha Transportation System; Sysmex, Kobe, Japan). The absolute count of a leukocyte subtype was calculated as the product of its respective differential percentage and total leukocyte count.

Serum creatinine concentrations were measured enzymatically using an Autoanalyzer (AU 5200, Olympus, Tokyo, Japan) and cystatin C was measured by latex immunoassay using a commercially available kit (IatroCys-C, Mitsubishi Chemical Medience, Tokyo, Japan). The creatinine-based estimated glomerular filtration rate (eGFR) was calculated using the equation recommended by the Japanese Society for Nephrology [17].

Data were presented as mean ± SD unless otherwise stated. Due to deviation from normal distribution, insulin, HOMA-IR, hsCRP, TNF-*α*, and IL-6 were logarithmically transformed for analysis. Differences between 2 groups were analyzed by* t*-test and frequencies of conditions by Chi-square tests. Differences among 3 groups were analyzed using analysis of variance. When *P* values in analysis of variance were *P* < 0.05, Bonferroni's multiple comparison procedure was performed. Correlations of PP were evaluated by Pearson's correlation analysis. Stepwise multiple regression analyses were performed to further identify the most significant variables contributing to the variation of PP. Potential confounders were forced into the model and standardized *β* coefficients were calculated. The explanatory power of the model was expressed as adjusted *R* ^2^ values. A two-tailed *P* < 0.05 was considered statistically significant. All calculations were performed with SPSS system 15.0 (SPSS Inc., Chicago, IL).

## 3. Results

As previously reported [14], participants were relatively healthy, community-living elderly women. PP averaged 59 ± 12 mmHg (Table 1). Of 150 women, 97 women (64.7%) had hypertension (52 women on antihypertensive medication and 45 women with SBP/DBP ≧ 140/90 mmHg without medication). High PP (≧65 mmHg) was found in 38 women (25.3%).

In univariate analysis (Table 1), PP was strongly and positively associated with age, SBP, and DBP. PP was also positively associated with log hsCRP, log TNF-*α*, and log IL-6. In addition, it showed positive associations with leukocyte, neutrophil, and monocyte count. Further, serum cystatin C was positively and eGFR was inversely associated with PP. After controlling for age (Table 1), associations with HDL cholesterol, adiponectin, and PAI-1 became significant. Associations with inflammatory markers except for log IL-6 remained significant. Associations with serum cystatin C and eGFR did not attain statistical significance.

We have done multiple regression analysis which included age and all variables that showed significant associations with PP after adjustment for age (partial correlation analysis) in Table 1. As shown in model A of Table 2, neutrophil count and log TNF-*α* emerged as determinants of PP independent of SBP and age (*R* ^2^ = 0.772). Results were the same in multiple regression analysis which included all inflammatory variables measured in this study (log hsCRP, log TNF-*α*, log IL-6, neutrophil, and monocyte count) as independent variables (Table 2, model B). Results in model A were confirmed in multiple regression analysis which included all variables that showed significant associations with PP in univariate analysis (model C); that is, neutrophil count and TNF-*α* were significant predictors of PP independent of hsCRP, IL-6, monocyte count, PAI-1, HOMA-IR, HDL cholesterol. and adiponectin.

Elderly women with high PP (≧65 mmHg) were older (Table 3) and had higher proportion of those on antihypertensive medications than those without it (66.0% versus 24.0%; *P* < 0.001). All women with high PP had hypertension whereas 59 out of 112 women with PP < 65 mmHg had hypertension (53.0%; *P* < 0.001). Elderly women with high PP had higher log hsCRP, log TNF-*α*, log IL-6, leukocyte, neutrophil, and monocyte count. In addition, elderly women with high PP had higher serum cystatin C and lower HDL-cholesterol than did women without high PP. After adjustment for age, differences remained significant in SBP, DBP, adiponectin, log hsCRP, neutrophil, and leukocyte count (data not shown).

## 4. Discussion

The present studies have demonstrated that brachial PP was associated with serum cystatin C, a sensitive marker of kidney function, than creatinine [18] and a broad range of inflammatory markers including log hsCRP, log TNF-*α*, log IL-6, peripheral monocyte, neutrophil, and leukocyte count in community-living elderly women. Among these variables, TNF-*α* and neutrophil count emerged as independent determinants of brachial PP after controlling for known confounders for PP. However, there was no association between PP and known risk factors for type 2 diabetes including adiponectin and HOMA-IR. We confirmed previous findings that PP is strongly associated with age and SBP [19, 20]. It is worthy to note that these findings were observed in community-living elderly women who had fewer indicators of disease, such as a low BMI, hypoalbuminemia, and hypocholesterolemia, which are usually considered a hallmark of malnutrition and frailty.

There is some evidence that increases in PP may stimulate inflammation. In humans, PP is positively associated with increased production of the reactive oxygen species [21], which, in turn, can stimulate inflammatory signaling pathways [22]. We confirmed previous findings in healthy middle-aged men that peripheral PP is correlated with inflammatory markers including CRP levels [19, 20, 23] and extended that, in elderly women, serum TNF-*α* and neutrophil count were correlated with PP independently of hsCRP.

Although TNF-*α* is an inflammatory cytokine produced mainly by monocytes and macrophages, TNF-*α* produced by adipose tissue may play an important role in obesity-associated insulin resistance and diabetes [24]. In the Insulin Resistance Atherosclerosis Study [25], circulating levels of TNF-*α* were elevated in individuals with impaired glucose tolerance and type 2 diabetes mellitus. In addition, increased TNF-*α* levels were predominantly associated with insulin resistance [25]. In our study of elderly women, TNF-*α* was correlated with PP independently of neutrophil count and hsCRP, hallmark of systemic inflammation. Taken together, these findings suggest that TNF-*α*- may be a biomarker of insulin resistance rather than systemic inflammation in our elderly women. It is probable that locally produced TNF-*α* may act synergistically with circulating TNF-*α* on fatty and muscular tissues to induce insulin resistance although the serum levels of TNF-*α* found in the present study were relatively low and circulating TNF-*α* may not be biologically active at such low concentration. There is one study which reported that TNF-*α* system is activated in accordance with PP in normotensive type 1 diabetes mellitus [26], in which TNF-*α* system is a reflection not of insulin resistance but of inflammation.

As previously reported in postmenopausal women with hypertension [27], an independent association of brachial PP with neutrophil count in our elderly women may be in accordance with the findings of a recent meta-analysis [28], which demonstrated that total WBC count as well as total granulocyte (and subset neutrophil) as well as lymphocyte but not monocyte count were significantly associated with type 2 diabetes. We, therefore, speculated that neutrophil count might better serve as a biomarker of systemic inflammation than hsCRP in our community-living elderly women. In postmenopausal women with hypertension [27], no inflammatory markers other than total and differentiated leukocyte count have been evaluated.

Previous studies have reported positive associations between CRP and direct and indirect measures of arterial stiffness [19, 20, 26, 29, 30], but most these studies analyzed single biomarkers or biomarkers for a single pathway. The present study examined associations between PP and a broad range of inflammatory markers and found a significant association of PP with TNF-*α* independent of hsCRP. Although it is known that TNF-*α* stimulates the synthesis of CRP in the liver, correlation coefficient between circulating levels of TNF-*α* and CRP is 0.173 in the present study and 0.27 in a large, multiethnic population of the Insulin Resistance Atherosclerosis Study [25]. As mentioned above, TNF-*α* may be a biomarker of insulin resistance rather than systemic inflammation in the present study as well.

In a subanalysis of the Candesartan Antihypertensive Survival Evaluation in Japan (CASE-J) Trial [10], it was suggested that increased PP, reflecting increased arterial stiffness, may be both a cause and a consequence of microvascular dysfunction, leading to a vicious cycle in impaired glucose metabolism and arteriolosclerosis [10]. In the present study increased PP was associated with biomarkers of insulin resistance (TNF-*α*) and systemic inflammation (neutrophil count) in elderly women.

Several limitations must be acknowledged. The cross-sectional design did not allow causal relationship. The recruitment procedure may also have some potential impact on the results. As the participation was voluntary, women who pay more attention to health may be more likely to participate. Biochemical parameters and blood pressure were measured only once and there was no follow-up data. Finally, we did not have detailed drug information although many participants were on antipressure drugs. It is known that some antipressure drugs reduce PP (e.g., angiotensin-converting enzyme inhibitors) and others increase it (e.g., vasodilators) [31].

## 5. Conclusions

The present studies have demonstrated an independent association of higher PP with local and systemic low-grade inflammation in community-living elderly women and suggest that low-grade inflammation may be one of the confounders for the association between high PP and incident type 2 diabetes in elderly patients with hypertension.

## Figures and Tables

**Table 1:** Anthropometric and biochemical characteristics of 150 free-living elderly women studied and correlation coefficients of brachial pulse pressure.

	Mean ± SD	Pulse pressure	
		Simple	Partial
Age (years)	75.5 ± 8.2	0.375***	Adjusted
BMI (kg/m^2^)	22.4 ± 2.8	−0.051	0.034
Body fat percentage (%)	32.8 ± 6.8	0.078	0.149
Systolic blood pressure (mmHg)	143 ± 19	0.841***	0.846***
Diastolic blood pressure (mmHg)	84 ± 10	0.335***	0.367***
Pulse pressure (mmHg)	59 ± 12	1.000	1.000
Plasma glucose (mg/dL)	86 ± 9	0.057	0.062
Insulin (*μ*U/mL)	5.4 ± 4.0	−0.017	−0.003
log insulin	0.66 ± 0.23	−0.017	0.026
HOMA-IR	1.18 ± 0.97	−0.001	−0.011
log HOMA-IR	−0.012 ± 0.25	−0.001	0.036
Total cholesterol (mg/dL)	221 ± 30	−0.059	−0.003
HDL-cholesterol (mg/dL)	67 ± 15	−0.136	−0.175*
LDL-cholesterol (mg/dL)	130 ± 28	0.029	0.103
Triglyceride (mg/dL)	118 ± 65	0.026	−0.015
Serum uric acid (mg/dL)	4.7 ± 1.0	0.022	0.078
Serum creatinine (mg/dL)	0.72 ± 0.15	0.141	0.027
Cystatin C (mg/L)	0.84 ± 0.19	0.312***	0.133
eGFR (mL/min/1.73 m^2^)	62 ± 13	−0.184*	−0.014
Leptin (ng/mL)	8.8 ± 5.8	0.026	0.112
Adiponectin (*μ*g/mL)	16.2 ± 7.4	−0.003	−0.193*
hsCRP (*μ*g/dL)	211 ± 355	0.266**	0.198*
Log hsCRP	1.88 ± 0.60	0.266**	0.239**
TNF-*α* (pg/mL)	2.25 ± 1.05	0.279***	0.274**
Log TNF-*α*	0.30 ± 0.21	0.279***	0.258**
PAI-1 (ng/mL)	28.3 ± 10.5	0.069	0.256**
IL-6 (pg/mL)	4.69 ± 5.95	0.211*	0.111
Log IL-6	0.47 ± 0.39	0.211*	0.154
Neutrophils (×10^3^/*μ*L)	3.27 ± 1.09	0.165*	0.289***
Lymphocytes (×10^3^/*μ*L)	2.03 ± 0.59	0.065	0.109
Monocytes (×10^3^/*μ*L)	0.31 ± 0.10	0.252**	0.244**
Leukocytes (×10^3^/*μ*L)	5.79 ± 1.39	0.188*	0.293***
Hemoglobin (g/dL)	12.9 ± 1.1	−0.120	−0.011

BMI: body mass index, HOMA-IR: homeostasis model assessment of insulin resistance, eGFR: estimated glomerular filtration rate, hsCRP: high-sensitivity C-reactive protein, TNF-*α*: tumor necrosis factor-*α*, PAI-1: plasminogen activator inhibitor-1, and IL-6: interleukin-6. *P < 0.05, **P < 0.01, and ***P < 0.001.

**Table 2:** Stepwise multiple regression analysis for pulse pressure as a dependent variable in community-dwelling elderly women.

	Standardized *β*	*P* value	Cumulative *R* ^2^
Model A			
Systolic blood pressure	0.775	<0.001	0.728
Age	0.140	0.001	0.749
Neutrophil count	0.114	0.006	0.763
Log TNF-*α*	0.110	0.009	0.772
Model B			
Systolic blood pressure	0.775	<0.001	0.728
Age	0.140	0.001	0.749
Neutrophil count	0.114	0.006	0.763
Log TNF-*α*	0.110	0.009	0.772
Model C			
Systolic blood pressure	0.775	<0.001	0.728
Age	0.140	0.001	0.749
Neutrophil count	0.114	0.006	0.763
Log TNF-*α*	0.110	0.009	0.772

Model A included age and all variables that showed significant associations with PP in partial correlation analysis in Table 1 as independent variables: SBP, HDL-cholesterol, adiponectin, log hsCRP, log TNF-*α*, PAI-1, neutrophil, monocyte, and leukocyte count. Model B included all inflammatory variables measured in this study (log hsCRP, log TNF-*α*, log IL-6, neutrophil, and monocyte count) as independent variables. Model C included all variables that showed significant associations with PP in univariate analysis.

**Table 3:** Anthropometric, biochemical, and hematological characteristics of elderly women with high pulse pressure (≧65 mmHg).

	PP < 65 mmHg	PP ≧ 65 mmHg	*P* value
	*n* = 112	*n* = 38	
Age (years)	74.3 ± 8.4	78.9 ± 6.5	0.001
BMI (kg/m^2^)	22.5 ± 2.8	22.0 ± 2.8	0.404
Body fat percentage (%)	32.5 ± 6.8	33.6 ± 7.1	0.417
Systolic blood pressure (mmHg)	136 ± 14	163 ± 15	0.000
Diastolic blood pressure (mmHg)	83 ± 10	89 ± 11	0.003
Pulse pressure (mmHg)	53 ± 8	75 ± 9	0.000
Plasma glucose (mg/dL)	86 ± 10	86 ± 8	0.736
Insulin (*μ*U/mL)	5.5 ± 4.0	5.0 ± 3.9	0.511
HOMA-IR	1.2 ± 1.0	1.1 ± 0.9	0.446
Total cholesterol (mg/dL)	223 ± 31	216 ± 27	0.238
HDL-cholesterol (mg/dL)	69 ± 16	63 ± 13	0.026
LDL-cholesterol (mg/dL)	130 ± 30	131 ± 23	0.837
Triglyceride (mg/dL)	120 ± 70	113 ± 48	0.556
Serum uric acid (mg/dL)	4.7 ± 1.0	4.8 ± 1.1	0.462
Serum creatinine (mg/dL)	0.71 ± 0.14	0.75 ± 0.18	0.185
Cystatin C (mg/L)	0.81 ± 0.16	0.94 ± 0.25	0.005
eGFR (mL/min/1.73 m^2^)	63 ± 12	60 ± 16	0.204
Leptin (ng/mL)	8.8 ± 5.9	8.9 ± 5.5	0.984
Adiponectin (*μ*g/mL)	16.5 ± 7.9	15.2 ± 5.8	0.300
hsCRP (*μ*g/dL)	171 ± 286	328 ± 491	0.069
Log hsCRP	1.81 ± 0.57	2.11 ± 0.61	0.007
TNF-*α* (pg/mL)	2.12 ± 0.97	2.63 ± 1.17	0.009
Log TNF-*α*	0.28 ± 0.21	0.38 ± 0.20	0.014
PAI-1 (ng/mL)	27.8 ± 10.3	29.7 ± 11.0	0.329
IL-6 (pg/mL)	4.20 ± 5.46	6.13 ± 7.09	0.084
Log IL-6 (pg/mL)	0.43 ± 0.37	0.58 ± 0.42	0.049
Neutrophils (×10^3^/*μ*L)	3.12 ± 0.97	3.72 ± 1.31	0.003
Lymphocytes	2.02 ± 0.57	2.06 ± 0.67	0.716
Monocytes (×10^3^/*μ*L)	0.29 ± 0.09	0.34 ± 0.11	0.011
Leukocytes (×10^3^/*μ*L)	5.61 ± 1.32	6.31 ± 1.48	0.007
Hemoglobin (g/dL)	13.0 ± 1.1	12.7 ± 1.1	0.094

Data are mean ± SD. Abbreviations are the same as in Table 1.

## Abbreviations

CVD: Cardiovascular disease
DBP: Diastolic blood pressure
eGFR: Estimated glomerular filtration rate
hsCRP: High-sensitivity C-reactive protein
HOMA-IR: Homeostasis model assessment
IL-6: Interleukin-6
LPL: Lipoprotein lipase
PAI-1: Plasminogen activator inhibitor-1
PP: Pulse pressure
SBP: Systolic blood pressure
TNF-*α*: Tumor necrosis factor-*α*.
